# Supplementary material for: Bovine leukemia virus encoded blv-miR-b4-3p microRNA is associated with reduced expression of anti-oncogenic gene in vivo
Source: PLoS One. 2023 Feb 2;18(2):e0281317. doi: 10.1371/journal.pone.0281317 (PMC9894392; doi:10.1371/journal.pone.0281317)
Supplement: S1 Data — (PDF) [file pone.0281317.s001.pdf]

PLOTTED and ANALYZED

An ID	PXDN N0_promedio	HK GEOM.MEAN	Target relative to HK	negLOG transform	**LOG trans**
6462	2,29906E-10	5,5583E-05	4,13626E-06	5,383	**-5,38**
6493	6,34186E-11	3,88017E-05	1,63443E-06	5,787	**-5,79**
5830	1,05389E-10	6,89991E-05	1,52739E-06	5,816	**-5,82**
5841	7,14202E-11	6,16868E-05	1,15779E-06	5,936	**-5,94**
6021	2,91797E-11	4,29396E-05	6,79551E-07	6,168	**-6,17**
6097	3,59387E-11	4,36295E-05	8,23725E-07	6,084	**-6,08**

An ID	miR29a NO_promedio	HK GEoM.MEAN	Target relative to HK	negLOG transform	**LOG trans**
6462	2,79858E-11	5,5583E-05	5,03496E-07	6,298	**-6,30**
6493	1,35654E-11	3,88017E-05	3,4961E-07	6,456	**-6,46**
5830	2,02154E-11	6,89991E-05	2,92981E-07	6,533	**-6,53**
5841	2,1726E-11	6,16868E-05	3,52198E-07	6,453	**-6,45**
6021	4,65055E-11	4,29396E-05	1,08304E-06	5,965	**-5,97**
6097	3,08945E-11	4,36295E-05	7,08111E-07	6,150	**-6,15**

An ID	miRb4_N0_promedio	HK GEoM.MEAN	Target relative to HK	negLOG transform	**LOG trans**
6462		5,5583E-05			
6493		3,88017E-05			
5830		6,89991E-05			
5841	3,44922E-10	6,16868E-05	5,5915E-06	5,252	**-5,25**
6021	5,94706E-10	4,29396E-05	1,38498E-05	4,859	**-4,86**
6097	1,12851E-09	4,36295E-05	2,58657E-05	4,587	**-4,59**

An ID	HBP1 N0_prom	HK GEOM_MEAN	Target relative to HK	negLOG transform	**LOG trans**
6462	1,31058E-05	0,000754129	0,017378762	1,760	**-1,76**
6493	1,47444E-05	0,000702627	0,020984694	1,678	**-1,68**
5830	1,55539E-05	0,000747331	0,02081258	1,682	**-1,68**
5841	1,6124E-05	0,000623653	0,025854193	1,587	**-1,59**
6021	8,99037E-06	0,000525592	0,017105223	1,767	**-1,77**
6097	1,62895E-05	0,000638435	0,025514768	1,593	**-1,59**

An ID	BLVpol_N0_AVE		N0 target: HK geommea	log_transf	**LOG trans**
5841	1,20501E-08	6,16868E-05	0,000195343	-3,709203	**-3,71**
6021	8,20464E-08	4,29396E-05	0,001910738	-2,718798779	**-2,72**
6097	5,06049E-08	4,36295E-05	0,001159878	-2,935587516	**-2,94**
