# Supplementary material for: Bovine leukemia virus encoded blv-miR-b4-3p microRNA is associated with reduced expression of anti-oncogenic gene in vivo
Source: PLoS One. 2023 Feb 2;18(2):e0281317. doi: 10.1371/journal.pone.0281317 (PMC9894392; doi:10.1371/journal.pone.0281317)
Supplement: Attachment — Submitted filename: rev. res. for PONE-D-22-32408_ PlosOne Dec 2022.docx [file pone.0281317.s002.docx]

The authors of the article entitled “Bovine leukemia virus encoded blv-miR-b4-3p microRNA is associated with reduced expression of anti-oncogenic gene in vivo” (#PONE-D-22-32408) did a good research job.

But…. you only wrote P values for Figure 1, D (PXDN mRNA), but you must write for the rest as well (Figure 1, A (BLV-pol), B (viral blv-miR-b4-3p), C (host miR-29a), and E (HBP-1 mRNA)), since the graphical difference is not always so obvious.

About the methods: Why didn't you measure DNA purity by the ratio of absorbance at 260/280 nm? This is one of the important indicators of DNA purity.

**Minor suggestions:**

In the fourth sentence of the abstract: “… host ́s …” ─ should be “… host …”.

In the last sentence of the abstract: “… BLV-encoding miRNAs…” ─ don't generalize to everything BLV-encoding miRNAs, but only blv-miR-b4-3p: It will be more correct.

Line 41: “… RNA virus” ─ does not correspond to the adhered classification, instead you should write “Deltaretrovirus genus”. If you want to emphasize that this is the RNA virus, then it is better to write additionally: “ According to the Baltimore classification system, BLV is the +ssRNA virus belonging to Group IV” [Baltimore D. Expression of animal virus genomes. Bacteriol Rev. 1971;35(3):235–41].

Lines 45: “… novo …” ─ should be “… *de novo …”*

Lines 45-46*:* Redundant information: “The novo synthesis of genomic viral RNA” and “viral transcripts” ─ rephrase this sentence.

Lines 48: “…persist…” ─ should be “… persists …”.

Lines 57: “...host ́s…” ─ should be “… host …”.

Lines 57-61: Where is the reference to these two sentences?

Lines 64: “… mRNA …” ─ should be “… mRNAs, …”.

Line 248: “… that BLV-encoding miRNAs might be involved in BLV tumorigenesis during natural infection” ─ I think that herein it is more correct not to focus on all BLV-encoding miRNAs, but only on blv-miR-b4-3p.
